# 24-month outcomes of XEN45 gel implant versus trabeculectomy in primary glaucoma

**DOI:** 10.1371/journal.pone.0256362

**Published:** 2021-08-19

**Authors:** Boonsong Wanichwecharungruang, Nitee Ratprasatporn

**Affiliations:** 1 Department of Ophthalmology, Department of Medical Services, Ministry of Public Health of Thailand, Rajavithi Hospital, Bangkok, Thailand; 2 Department of Ophthalmology, Priest Hospital, Bangkok, Thailand; University of Missouri-Columbia, UNITED STATES

## Abstract

**Purpose:**

To compare the efficacy and safety profiles of XEN implant versus trabeculectomy as a surgical intervention for primary glaucoma

**Methods:**

A retrospective cohort study of mild to moderate stage glaucoma patients, who had undergone either XEN implantation or trabeculectomy with adjunctive mitomycin C, was performed in a tertiary eye center

**Results:**

Fifty-seven eyes for XEN implant and 57 eyes for trabeculectomy with medically uncontrolled glaucoma were included. Preoperative IOP was 16–33 mmHg. Visual field mean deviation was -9.11±6.93 dB in XEN group, and -9.67±5.06 dB in trabeculectomy group (p = 0.195). At the 24-month timepoint, mean IOP was reduced from 21.6±4.0 to 14.6±3.5 mmHg (32.4% reduction) in the XEN group (p<0.001), and from 22.5±5.8 to 12.5±4.1 mmHg (44.4% reduction) in the trabeculectomy group (p<0.001). Final IOP in XEN was significantly higher than trabeculectomy (p = 0.008) with lesser mean IOP percentage reduction at month 24 (p = 0.045). Mean number of medications was reduced from 2.2±1.4 to 0.5±0.7 in XEN group (p<0.001), and from 2.4±0.7 to 0.8±1.3 in trabeculectomy group (p<0.001). Final number of medications was not different between the groups (p = 0.225). Surgical success was comparable between XEN and trabeculectomy group. Overall success was 71.4% vs. 73.3% (p = 0.850), and complete success was 62.9% vs. 62.2% (p = 0.954), respectively. XEN had lower rate of numerical hypotony than trabeculectomy. No serious complication occurred in either procedure group.

**Conclusion:**

At 24 months, XEN showed a rate of success comparable to that of trabeculectomy. Although XEN had a higher final IOP than trabeculectomy, XEN achieved 32% IOP reduction, and achieved final IOP in mid-teen level. No serious complication occurred in either group. XEN can be applied for treatment of mild to moderate stages of glaucoma in Southeast Asian patients.

## Introduction

Glaucoma is a leading cause of irreversible blindness worldwide, and Asians and Africans account for a large proportion of its global incidence [1]. Asia has the highest prevalence of primary angle-closure glaucoma (PACG), while Africa has the greatest prevalence of primary open-angle glaucoma (POAG) [1]. Glaucoma is a chronic progressive optic neuropathy characterized by typical optic nerve changes and corresponding visual field defects. Damage or changes to the trabecular meshwork limit the aqueous humor outflow, leading to high intraocular pressure (IOP), which is an important risk factor for disease progression [2]. The patient’s sight can be saved by proper management, including medications, and laser treatment. In the event that these treatments are unsuccessful, surgery is usually employed to control IOP. Trabeculectomy has been the standard procedure for decades [3], and evidence-based studies have demonstrated its long-term efficacy and safety [4,5], however, some serious complications can occur, such as malignant glaucoma, bleb-related infection and expulsive choroidal hemorrhage, with devastating consequences [6–8].

Recently, many novel ophthalmic surgical instruments and implants have been employed in glaucoma surgery. These newer procedures, so-called “minimally invasive glaucoma surgery (MIGS)”, offer a shorter operative time and learning curve, and they appear to be less invasive than trabeculectomy. XEN implant (XEN45 Glaucoma gel implant; Allergan, Irvine, California, USA), a form of MIGS, has been available in clinical practice for a few years; however, safety and effectiveness data are limited in Southeast Asian patients. We conducted a comparative study of the surgical outcomes of XEN and trabeculectomy in a cohort of Southeast Asian patients.

## Patients and methods

This retrospective cohort study was conducted in a tertiary eye care hospital. Age-matched (age within 5 years between the two groups) and diagnosis-matched (either POAG or PACG) primary glaucoma patients who underwent XEN implantation or trabeculectomy between February 2018 and December 2019 were enrolled for chart review. The study protocol was approved by the ethics committee (IRB) of Rajavithi Hospital, and written informed consent was read and signed by all participants, who underwent complete eye examination including visual acuity, auto-refraction, slit-lamp examination, Goldmann applanation tonometry, gonioscopy, cup/disc ratio and fundus ophthalmoscopy. The individual in this manuscript has given written informed consent (as outlined in PLOS consent form) to publish these case details. Visual field, central corneal thickness (CCT) and OCT were also obtained. Enrolled patients were those that consecutively underwent the procedures within the specified period.

Definition of POAG included those who had normal anterior segment, IOP > 21 mmHg prior to surgery, gonioscopic open angle in all quadrants, glaucomatous optic disc changes, and/or corresponding visual field defect. Definition of PACG included initial IOP > 21 mmHg prior to surgery, shallow anterior chamber, gonioscopic Shaffer’s grade ≤ 2 in 2 or more quadrants, and glaucomatous optic disc changes and/or corresponding visual field defect.

Inclusion criteria included primary glaucoma, and age ≥ 18 years old. Phakic or pseudophakic patients with history of uneventful phacoemulsification performed at least 6 months prior to their glaucoma surgeries were eligible, as were those who had previous laser iridotomy, iridoplasty, and trabeculoplasty (argon laser or selective laser). Both eyes could be included if they met the inclusion criteria, and the surgery for each eye was performed ≥30 days apart.

Exclusion criteria were any other glaucoma apart from POAG or PACG, previous intraocular glaucoma surgery, presence of pathologies involving the target quadrant of the conjunctiva, corneal opacity which might obscure XEN, presence of intraocular silicone oil, presence of vitreous in the anterior chamber, clinically significant or active infection or inflammation, active or previous eye disease that could interfere with study results, established or suspected sensitivity/allergy to medications or any of the device components required for surgery (including anesthesia), and patients with limited follow-up.

### Perioperative procedure

All XEN implantations were performed under local anesthesia by a senior glaucoma specialist (B.W.). For trabeculectomy, the procedure was performed by either a senior instructor (B.W.) or a glaucoma clinical fellow under the supervision of a senior instructor (B.W.).

For stand-alone XEN implantation, the supero-nasal conjunctiva was marked 3 mm from the limbus. Xylocaine 2%, 0.05 mL mixed with mitomycin-C (MMC) 20 microgram 0.05 mL (400 microgram per mL) was injected into the subconjunctiva at the marked area to enlarge the subconjunctival space. Clear corneal main-port and side-port incisions were created temporally, and dispersive viscoelastic was injected to fill the anterior chamber. Preloaded injector was inserted through the main incision aiming toward the superior-nasal quadrant, after which the needle was advanced through the sclera into the subconjunctival space. After the surgeon had confirmed the visualization of the needle tip bevel within the subconjunctival space, a stent was introduced. A gonio lens was used prior to or after the introduction of the XEN at the discretion of the surgeon. The stent was ideally anticipated to be 1 mm in the anterior chamber, 2 mm in the scleral tunnel, and 3 mm in the subconjunctival space, and the injector was then removed. The subconjunctival XEN part was tested to ensure it was freely mobile, and the viscoelastic was removed once the implant was properly positioned; adjustment of the implant was performed if necessary. The anterior chamber was reformed with a balanced salt solution, and the subconjunctival bleb formation was checked. All incisions were sealed by hydration at the conclusion of the surgery.

For combined XEN implantation with phacoemulsification (phaco+XEN), we first performed phacoemulsification with intraocular lens implantation under topical anesthesia. XEN implantation was then performed in the same manner as described above.

For trabeculectomy, fornix-based conjunctival incision was performed, and a triangular scleral flap was created. Subconjunctival MMC 400 microgram per mL was applied for 4 minutes after which BSS rinsing was carried out. Paracentesis, internal sclerectomy with Kelly Descemet’s Membrane Punch and peripheral iridectomy were then performed after which the scleral flap was sutured and fluorescein dye was applied to check for appropriate aqueous outflow. The conjunctiva was finally sutured, and the stitches were buried.

Postoperative treatment regimen included topical steroid (prednisolone acetate 1%) every 2 hours for 1 week, and QID for the next 3 weeks; this treatment tapered off gradually. Steroids were adjusted thereafter according to the postoperative degree of inflammation. Topical antibiotic (fluoroquinolone) was applied QID for 4 weeks.

When adjunctive IOP-lowering treatment was required after the surgery, laser suture lysis, reintroducing glaucoma medications, and/or bleb needling could be performed. Needling of the bleb was documented as a post-operative procedure and was not counted as a glaucoma-related secondary surgical intervention (SSI) or adverse event (AE) [9,10]. The use of additional MMC injection at the time of needling was at the discretion of the surgeon.

### Assessments

Demographic data, age, gender, diagnosis (POAG or PACG), monocular best-corrected visual acuity (VA; measured in Snellen and converted to decimals), pachymetry (central corneal thickness), ophthalmoscopy (cup-to-disc ratio), visual field (mean deviation on standard automated perimetry (SAP) based on SITA Standard 24–2 protocol), mean medicated preoperative IOP (measured by Goldmann applanation tonometry) and mean preoperative medication count, were assessed at baseline. Date of surgery, surgery type (stand-alone vs combined), implant location, use of antifibrotic agents (dose and timing) and intraoperative complications were recorded intraoperatively. Postoperative follow-ups were performed at day 1, weeks 1 and 2, and months 1, 3, 6, 9, 12, 18 and 24. Mean IOP and number of anti-hypertensive medications were assessed at each postoperative timepoint. Postoperative safety assessments included slit-lamp biomicroscopy, AEs, and requirement for needling and SSI at each postoperative follow-up. Mean IOP reduction, number of medications, mean deviation, cup-to-disc ratio, success rates and safety profiles were analyzed at 3, 6, 12, 18 and 24-month follow-up timepoints.

### Outcomes and analyses

The main outcomes measured were mean IOP reduction and the number of IOP-lowering medications needed at each timepoint after XEN implantation or trabeculectomy.

Other outcomes included proportions of eyes achieving overall success and complete success, proportion of eyes that required needling, the amount of needling required per eye, AEs (by counts and percentages), and subgroup analysis of mean IOP reduction and the number of IOP-lowering medications in POAG and PACG eyes.

### Surgical success

The definitions of success and failure were as follows:

Overall success: eyes achieving ≥20% reduction in IOP from the preoperative baseline with or without medication;

Complete success: eyes achieving ≥20% reduction in IOP from the preoperative baseline without medication; and

Failure: eyes not fulfilling overall success criteria, or loss of light perception.

Postoperative IOP measurements and number of IOP-lowering medications were compared with preoperative values and were analyzed with paired t-test. Comparison between the XEN45 and trabeculectomy groups was performed with two-sided t-test, and descriptive statistics were performed for the summarization of all endpoints. Proportions and AEs were analyzed with Chi-square and Fisher’s exact test while subgroup and other outcomes were analyzed with two-sided t-test and Mann-Whitney U test. Failure was examined with Kaplan-Meier survival analysis, and *P* values <0.05 were considered to be statistically significant. Statistical analysis was performed with SPSS V.25 (SPSS Inc., Chicago, IL, USA).

## Results

A total of 118 eyes (61 for XEN implant and 57 for trabeculectomy) were recruited. However, 4 cases of XEN were excluded from the study due to loss to follow-up. Total 57 XEN cases were enrolled for analysis. Of these cases, there were combined phacoemulsification with intraocular lens and XEN implant (Phaco+XEN) implant in 4 eyes (7.0%). No combined cataract surgery and trabeculectomy was performed. Demographic data is shown in **Table 1**. Preoperative diagnosis was POAG, 73.7% in each group. Mean age, refraction, pre-operative IOP, mean number of IOP-lowering medications, CCT, axial length, cup-to-disc ratio and visual field MD were not significantly different in the two groups. The VA in the XEN group was better than in the trabeculectomy group (p = 0.001). Three patients in the XEN group and two patients in the trabeculectomy group had both eyes included in the study. Trabeculectomy was performed by a glaucoma fellow under the supervision of B.W. in 5 eyes.

**Table 1:** Baseline characteristics of XEN45 and trabeculectomy groups.

Variable	XEN45 (n = 57)	Trabeculectomy (n = 57)	p-value
Gender; n (%)			0.349*
Male	26 (45.6)	31 (54.4)	
Female	31 (54.4)	26 (45.6)	
Age (year)	70.4 ± 8.4	68.9 ± 8.6	0.334^†^
Ethnicity (Southeast Asian)	100%	100%	-
VA (decimal)	0.67 ± 0.23	0.50 ± 0.27	0.001^†^
Refraction (SE)	-0.52 ± 1.72	-0.89 ± 2.46	0.368^‡^
CCT (μm)	524.1 ± 13.0	527.2 ± 13.5	0.209^†^
Axial length (mm)	23.51 ± 0.88	23.65 ± 1.14	0.470^†^
Diagnosis; n (%)			1.000*
POAG	42 (73.7)	42 (73.7)	
PACG	15 (26.3)	15 (26.3)	
Lens status; n (%)			0.089*
Phakic	20 (35.1)	30 (52.6)	
Pseudophakic	37 (64.9)	27 (47.4)	
Diabetes; n (%)	13 (22.8)	16 (28.1)	0.519*
Prior laser iridotomy; n (%)	15 (26.3)	16 (28.1)	0.833*
Prior laser trabeculoplasty; n (%)	9 (15.8)	4 (7.0)	0.141*
Cup-to-disc ratio	0.73 ± 0.10	0.76 ± 0.13	0.076^†^
Mean deviation (dB)	-9.11 ± 6.93	-9.67 ± 5.06	0.195^‡^
Mean number of medications	2.2 ± 1.4	2.4 ± 0.7	0.434^†^
Mean IOP (mmHg)	21.6 ± 4.0	22.5 ± 5.8	0.379^†^

*Chi-square test

†Student’s t-test

‡Mann-Whitney U test.

### Intraocular pressure

**Fig 1:**
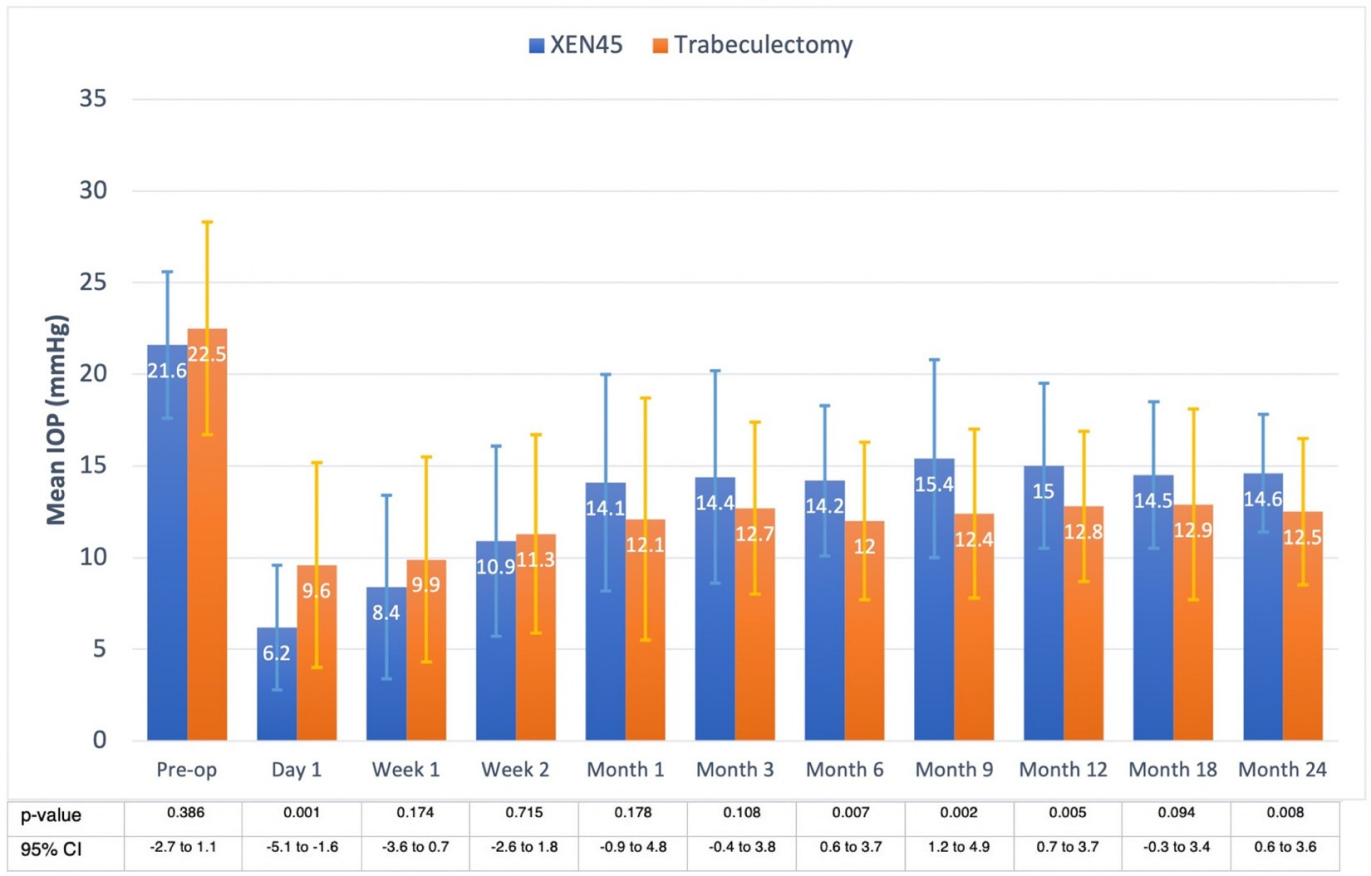
Mean intraocular pressure of the XEN implant and trabeculectomy groups. Preoperative IOP was comparable in the two groups. Postoperative IOPs were significantly different from month 6 onward except month 18. Final mean IOP was 14.6 mmHg in the XEN and 12.5 mmHg in the trabeculectomy group.

**Fig 2:**
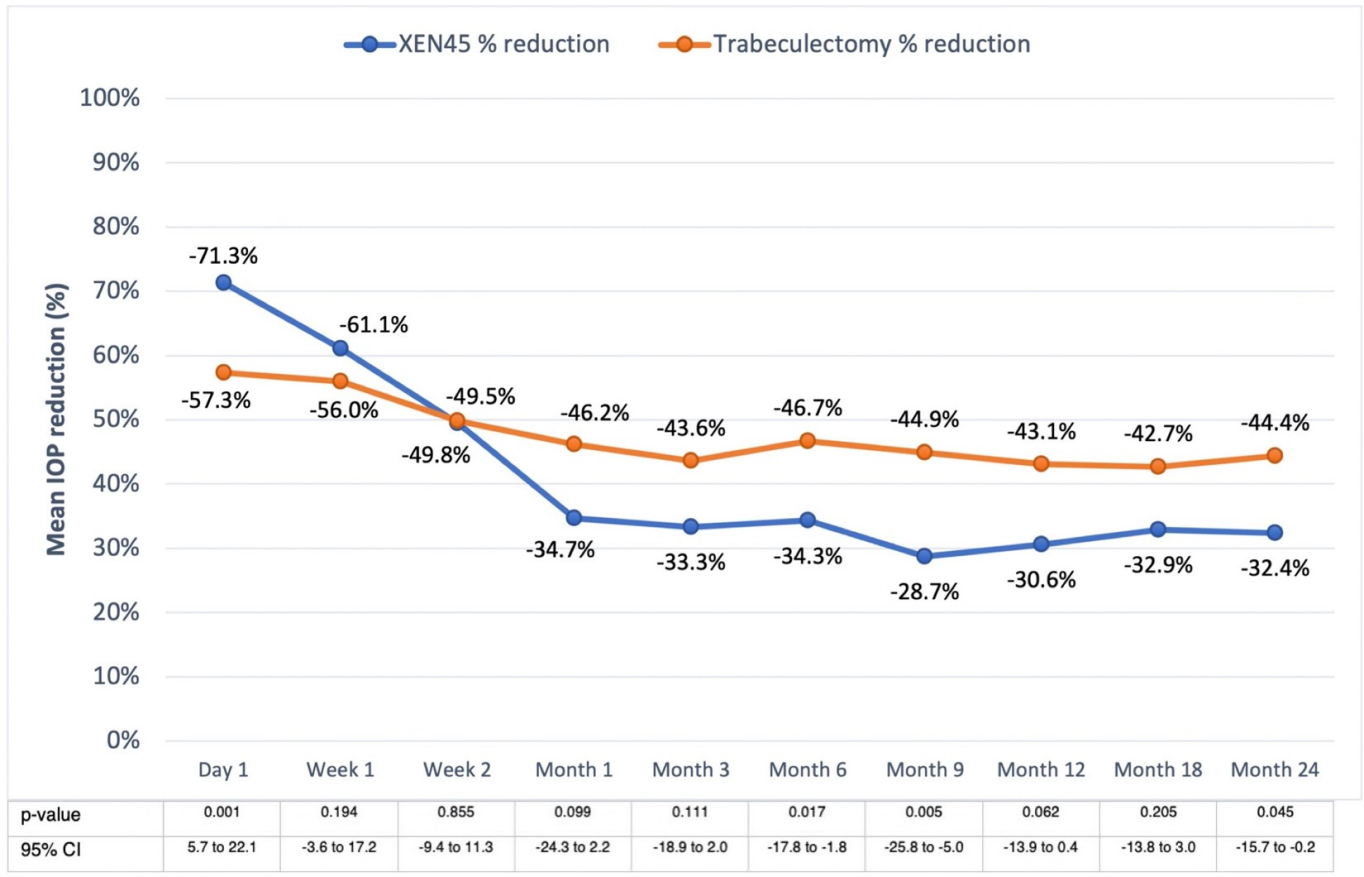
Mean intraocular pressure percent reduction of the XEN implant and trabeculectomy groups. Postoperative IOPs were significantly different on day 1, month 6, month 9 and month 24. Final mean IOP reduction was 32.4% in the XEN and 44.4% in the trabeculectomy group.

IOP was significantly lower in the XEN group at day 1 postoperatively (p = 0.001), and in the trabeculectomy group from months 6 to 24 (all p<0.01) except for month 18 (p = 0.094). Final IOP at month 24 was higher in the XEN45 than in the trabeculectomy group at 14.6 and 12.5 mmHg (p = 0.008), respectively. Each postoperative timepoint had a statistically significant reduction in IOP compared with baseline preoperative IOP in both groups (all p<0.001), **Fig 1**. Percentage of IOP reduction from month 6 onward ranged from 28.7–34.3% in the XEN group and 42.7–46.7% in the trabeculectomy patients. Comparing the percentage IOP reduction between the two groups, the XEN group achieved greater percentage IOP reduction on day 1 (p = 0.001), then, the percentage difference converged from week 1 to month 3 (all p>0.05) and eventually the trabeculectomy group achieved greater percentage IOP reduction on month 6 (p = 0.017), month 9 (p = 0.005) and month 24 (p = 0.045) but the difference was not found on month 12 (p = 0.062) and month 18 (p = 0.205). **Fig 2.**

### Number of medications

**Fig 3:**
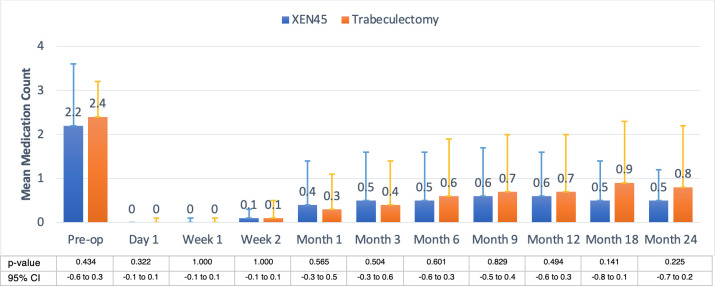
Mean number of medications between the XEN implant and trabeculectomy groups. Preoperative medications in XEN were comparable to trabeculectomy, at 2.2 versus 2.4. The postoperative numbers of medications were not different at any timepoint. The final numbers of medications were 0.5 in the XEN group and 0.8 in the trabeculectomy group.

The preoperative medication count in XEN patients was comparable to trabeculectomy counterparts, 2.2 vs. 2.4 (p = 0.434), and no difference was detected at any postoperative timepoint (all p>0.05). Each postoperative timepoint had a statistically significant reduction in the number of IOP-lowering medications compared with baseline preoperative values in both groups (all p<0.001). Final number of medications was 0.5 vs. 0.8, respectively (p = 0.225), **Fig 3.** The number of medications reported for all the visits were representative of the entire cohort.

### Surgical success

**Fig 4:**
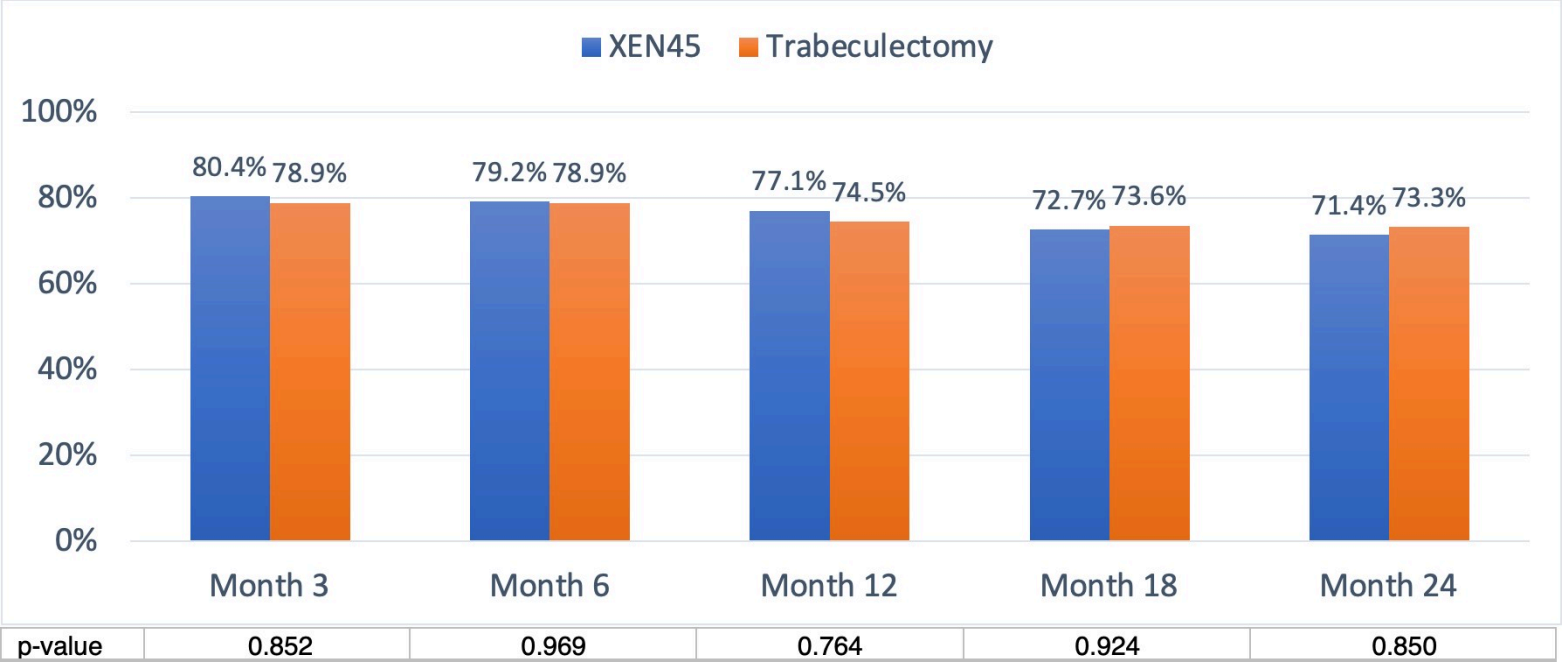
Overall success was defined as postoperative IOP reduced ≥ 20% from the preoperative baseline, with or without medications. Overall success of XEN implant and trabeculectomy were comparable at all timepoints. At month 24, the overall success was 71.4% and 73.4% respectively.

**Fig 5:**
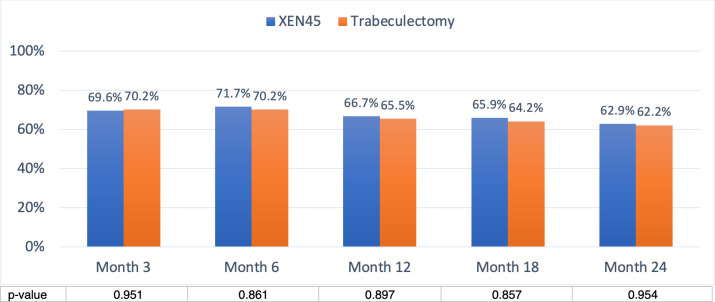
Complete success was defined as postoperative IOP reduced ≥ 20% from the preoperative baseline, without any medications. Complete success of XEN implant and trabeculectomy were comparable at all timepoints. At month 24, the complete success rates were 62.9% versus 62.2% respectively.

Overall success in the XEN group was 80.4%, 79.2%, 77.1%, 72.7% and 71.4% at 3, 6, 12, 18 and 24 months, respectively, and 78.9%, 78.9%, 74.5%, 73.6% and 73.3% at 3, 6, 12, 18 and 24 months respectively in the trabeculectomy group **(Fig 4)**. Overall success was similar in the two groups (all p>0.05). Complete success in the XEN group was achieved in 69.6%, 71.7%, 66.7%, 65.9% and 62.9% of cases at 3, 6, 12, 18 and 24 months, respectively, and 70.2%, 70.2%, 65.5%, 64.2% and 62.2% at 3, 6, 12, 18 and 24 months respectively in the trabeculectomy group **(Fig 5)**. Complete success was comparable in the two groups (all p>0.05).

### Survival analysis

**Fig 6:**
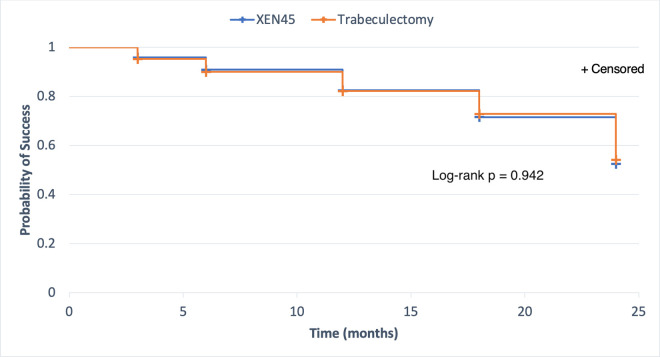
Kaplan-Meier curve showing the probability of achieving any success criteria in the XEN implant and trabeculectomy groups.

**Fig 6** is presented as a Kaplan-Meier survival estimate graph with right censoring. In the XEN group, 5% of eyes had failed (SE 1%) at month 3, 10% had failed (SE 2%) at month 6, 18% had failed (SE 3%) at month 12, 27% had failed (SE 4%) at month 18, and 46% had failed (SE 6%) at month 24. In the trabeculectomy group, 5% of eyes had failed (SE 1%) at month 3, 10% had failed (SE 2%) at month 6, 18% had failed (SE 3%) at month 12, 29% had failed (SE 4%) at month 18, and 48% had failed (SE 5%) at month 24. No difference in failure between the two groups was noted (Log-rank p = 0.942). Eyes that failed to meet success criteria at month 3, 6, 12 or 18 were considered as failure, even if later on they met the success criteria.

### Visual acuity

The XEN group had Snellen VA in decimals of 0.67±0.23 at baseline, and 0.64±0.19, 0.64±0.20, 0.67±0.19 and 0.66±0.17 at month 6, 12,18 and 24, respectively, and VA did not change significantly compared with preoperative VA in this group during the postoperative follow-up timepoints. Overall, no patient lost >2 Snellen lines equivalent VA in the XEN45 group. In the combined group (Phaco+XEN), VA improved from 0.45±0.16 to 0.53±0.23, 0.62±0.17, 0.62±0.17 and 0.60±0.08 at month 6, 12, 18 and 24, respectively; however, these results were not statistically significant.

In the trabeculectomy group, Snellen VA in decimals was 0.50±0.27 at baseline, and this changed to 0.49±0.26, 0.47±0.27, 0.44±0.28 and 0.43±0.25 at months 6, 12, 18 and 24, respectively. VA suffered a statistically significant deterioration from the baseline in the trabeculectomy group at month 24 (p = 0.037), but this was without clinical significance when translated into Snellen equivalent VA. Two patients lost >2 Snellen lines equivalent VA in the trabeculectomy group: one was related to the development of epiretinal membrane and cataract by month 12 postoperatively, and the other was related to branch retinal vein occlusion by month 12 postoperatively.

### Optic disc and visual field

Cup-to-disc ratio compared with baseline was stable in both groups throughout the postoperative follow-up timepoints. The XEN group had cup-to-disc ratio of 0.73±0.10 at baseline, and this changed to 0.73±0.09 at month 24, respectively. In the trabeculectomy group, cup-to-disc ratio was 0.76±0.13 at baseline, changing to 0.77±0.10 at month 24, respectively. No rapid progression of cup-to-disc ratio was detected in any patient.

Also, no statistically significant difference in MD was detected compared with baseline in the two groups throughout the postoperative follow-up timepoints. The XEN group had MD of -9.11±6.93 dB at baseline, and this altered to -9.99±6.54 dB, -9.77±6.65 dB, -9.71±6.14 dB and -9.84±6.41 dB at months 6, 12, 18 and 24, respectively. In the trabeculectomy group, MD was -9.67±5.06 dB at baseline, and this shifted to -9.71±7.60 dB, -10.67±8.12 dB, -11.12±6.03 dB and -11.51±8.50 dB at months 6, 12, 18 and 24, respectively. Rapid deterioration of MD, caused by hypotony maculopathy and choroidal detachment, was detected in one trabeculectomy patient at month 6. The MD of this patient was -13.01 dB at baseline, and this declined to -27.60 dB at the month 12 timepoint. All the visual fields data is complete for every patient at every time-point except for 2 participants in the XEN group at month 24, and 2 participants in the trabeculectomy group at month 24.

### Needling revision

In the XEN group, needling was required in 10 patients (17.5%) during the follow-up period. The mean number of needling events at 24 months was 0.21±0.49 with a range of 0 to 2, and the mean and median time to the first needling were 132±130 days and 89 days. 4 (40.0%) patients were successful after the needling while 6 (60.0%) patients failed and needed further medications or SSI. In the trabeculectomy group, needling was also required in 11 patients (19.3%) in the follow-up period. The mean number of needling events at 24 months was 0.23±0.50 with a range of 0 to 4. 8 (72.7%) patients were successful after the needling while 3 (27.3%) patients failed. Anti-glaucoma medications were started initially in both groups and if the IOP could not be controlled, needling was indicated. The mean and median time to the first needling were 98±93 days and 68 days. Although the number of patients requiring needling was not different in the two groups, the trabeculectomy patients required earlier needling than their XEN counterparts.

### Subgroup analysis between POAG and PACG

Subgroup analysis comparison of POAG (N = 42) and PACG (N = 15) patients in the XEN group was performed and the results are shown in **Table 2**. This information pertains to all patients in the cohort.

**Table 2:** Characteristics of XEN45 and trabeculectomy in POAG and PACG.

Variable	XEN45		p-value	Trabeculectomy		p-value
	POAG (n = 42)	PACG (n = 15)		POAG (n = 42)	PACG (n = 15)	
Gender; n (%)			0.611*			0.924*
Male	20 (47.6)	6 (40.0)		23 (54.8)	8 (53.3)	
Female	22 (52.4)	9 (60.0)		19 (45.2)	7 (46.7)	
Age (year)	70.2 ± 8.9	71.1 ± 7.4	0.728^†^	69.6 ± 9.0	67.1 ± 7.1	0.348^†^
VA (decimal)	0.66 ± 0.24	0.69 ± 0.20	0.650^†^	0.50 ± 0.26	0.52 ± 0.28	0.824^†^
Refraction (SE)	-0.60 ± 1.80	-0.30 ± 1.49	0.574^‡^	-0.89 ± 2.43	-0.92 ± 2.60	0.968^‡^
CCT (μm)	523.1 ± 13.8	526.9 ± 10.4	0.337^†^	533.2 ± 8.8	533.1 ± 10.8	0.897^†^
Axial length (mm)	23.51 ± 0.90	23.51 ± 0.84	1.000^†^	23.99 ± 0.92	23.13 ± 1.10	0.055^†^
Lens status; n (%)			0.642*			0.205*
Phakic	14 (33.3)	6 (40.0)		20 (47.6)	10 (66.7)	
Pseudophakic	28 (66.7)	9 (60.0)		22 (52.4)	5 (33.3)	
Cup-to-disc ratio	0.71 ± 0.12	0.74 ± 0.11	0.430^†^	0.78 ± 0.13	0.75 ± 0.16	0.435^†^
Mean deviation (dB)	-9.04 ± 6.56	-9.31 ± 8.13	0.897^‡^	-9.81 ± 4.98	-9.26 ± 5.43	0.722^‡^
Mean number of medications	2.3 ± 1.4	2.1 ± 1.2	0.559^†^	2.5 ± 0.7	2.2 ± 1.0	0.339^†^
Mean IOP (mmHg)	22.1 ± 3.6	20.3 ± 4.8	0.122^†^	22.8 ± 6.1	21.2 ± 4.9	0.202^†^
Mean IOP at month 3 (mmHg)	14.3 ± 5.5	14.7 ± 6.6	0.829^†^	13.0 ± 4.6	11.6 ± 5.2	0.314^†^
Mean IOP at month 6 (mmHg)	14.6 ± 4.1	13.0 ± 3.9	0.199^†^	12.1 ± 4.6	11.5 ± 3.6	0.642^†^
Mean IOP at month 12 (mmHg)	15.2 ± 4.2	14.4 ± 5.3	0.540^†^	12.7 ± 4.2	13.0 ± 3.9	0.828^†^
Mean IOP at month 18 (mmHg)	14.6 ± 3.1	14.2 ± 6.1	0.761^†^	13.1 ± 5.3	12.5 ± 5.3	0.739^†^
Mean IOP at month 24 (mmHg)	15.0 ± 3.5	13.3 ± 3.5	0.100^†^	12.5 ± 4.2	12.4 ± 3.3	0.927^†^
Mean number of medications at month 3	0.5 ± 1.1	0.6 ± 1.2	0.717^†^	0.4 ± 1.1	0.3 ± 0.7	0.659^†^
Mean number of medications at month 6	0.5 ± 1.0	0.5 ± 1.2	0.804^†^	0.7 ± 1.4	0.4 ± 0.7	0.120^†^
Mean number of medications at month 12	0.5 ± 1.0	0.7 ± 1.1	0.639^†^	0.8 ± 1.4	0.5 ± 0.7	0.119^†^
Mean number of medications at month 18	0.5 ± 0.9	0.5 ± 0.9	0.972^†^	0.9 ± 1.4	0.5 ± 0.9	0.113^†^
Mean number of medications at month 24	0.5 ± 1.0	0.5 ± 0.9	0.961^†^	0.9 ± 1.4	0.5 ± 0.7	0.166^†^
Overall success at month 3 (%)	80.5	80.0	0.968*	78.6	80.0	0.907*
Overall success at month 6 (%)	79.0	80.0	0.932*	78.6	80.0	0.907*
Overall success at month 12 (%)	79.0	73.3	0.677*	75.0	73.3	0.899*
Overall success at month 18 (%)	72.4	73.3	0.948*	73.7	73.3	0.979*
Overall success at month 24 (%)	70.0	73.3	0.829*	73.3	73.3	1.000*
Complete success at month 3 (%)	70.7	66.7	0.770*	69.1	73.3	0.755*
Complete success at month 6 (%)	71.1	73.3	0.868*	69.1	73.3	0.755*
Complete success at month 12 (%)	66.7	66.7	1.000*	65.0	66.7	0.908*
Complete success at month 18 (%)	65.5	66.7	0.939*	63.2	66.7	0.810*
Complete success at month 24 (%)	65.0	60.0	0.918*	63.2	60.0	0.831*

*Chi-square test

†Student’s t-test

‡Mann-Whitney U test.

Baseline characteristics including mean IOP and number of medications were similar in POAG and PACG patients. Mean IOP and number of medications were significantly lower than the baseline in both groups at all timepoints (all p<0.001). Mean IOP and mean number of medications were not statistically different in the POAG and PACG patients at all timepoints (all p>0.05). Overall success and complete success were comparable in the POAG and PACG patients at all timepoints (all p>0.05).

Subgroup analysis comparison of POAG (N = 42) and PACG (N = 15) patients in the trabeculectomy group was performed. Baseline characteristics including mean IOP and number of medications were similar in POAG and PACG patients. Mean IOP and number of medications were significantly lower than the baseline in both groups at all timepoints (all p<0.001). Mean IOP and mean number of medications were not statistically different in the POAG and PACG patients at all timepoints (all p>0.05). Overall success and complete success were comparable in the POAG and PACG patients at all timepoints (all p>0.05).

In terms of comparing XEN and trabeculectomy in the POAG group, baseline characteristics including mean IOP and number of medications were similar in both groups. Mean IOP and number of medications were significantly lower than the baseline in both groups at all timepoints (all p<0.001). Mean IOP was lower in the XEN group on postoperative day 1 (p = 0.002) and was lower in the trabeculectomy group from month 6 to 24 (all p<0.05) except month 18 (p = 0.139) and mean number of medications were not statistically different in both groups at all timepoints (all p>0.05). Overall success and complete success were comparable in both groups at all timepoints (all p>0.05).

Comparing XEN and trabeculectomy in the PACG group, baseline characteristics including mean IOP and number of medications were similar in both groups. Mean IOP and number of medications were significantly lower than the baseline in both groups at all timepoints (all p<0.001). Mean IOP was lower in the XEN group on postoperative day 1 (p = 0.041) and was lower in the trabeculectomy group on month 9 (p = 0.047) while other timepoints failed to show the IOP difference between the two groups (p>0.05). The mean number of medications were not statistically different in both groups at all timepoints (all p>0.05). Overall success and complete success were comparable in both groups at all timepoints (all p>0.05).

Of note, the surgery on five eyes in the trabeculectomy group were performed by a glaucoma fellow under the supervision of a senior instructor (B.W.). The final mean IOP (11.6±3.4 vs. 12.5±4.0 mmHg, p = 0.623), mean number of medications (0.8±1.1 vs 0.9±1.4, p = 0.895), overall success (80.0% vs 72.5%, p = 0.708) and complete success (60.0% vs 62.5%, p = 0.848) were similar when performed by a glaucoma fellow or by a senior instructor, respectively. However, the lack of difference may be due to the small number of patients operated by a glaucoma fellow.

### Subgroup analysis between phakic and pseudophakic patients

Subgroup analysis comparison of phakic (N = 20) and pseudophakic (N = 37) patients in the XEN group was performed and the results are shown in **S1 Table**. Four patients in the phakic group who underwent combined Phaco+XEN were excluded from this analysis.

Baseline characteristics including mean IOP and number of medications were similar in the two groups except pseudophakic patients are older than phakic patients (73.9 ± 7.0 vs. 64.1 ± 7.2, p<0.001). Mean IOP and number of medications were significantly lower than the baseline in both groups at all timepoints (all p<0.001). Mean IOP and mean number of medications were not statistically different in the phakic and pseudophakic patients at all timepoints (all p>0.05). Overall success and complete success were comparable in the phakic and pseudophakic patients at all timepoints (all p>0.05).

Subgroup analysis comparison of phakic (N = 30) and pseudophakic (N = 27) patients in the trabeculectomy group showed baseline characteristics including mean IOP and number of medications were similar in the two groups except pseudophakic patients are older than phakic patients (73.5 ± 7.8 vs. 64.8 ± 7.0, p<0.001). Mean IOP and number of medications were significantly lower than the baseline in both groups at all timepoints (all p<0.001). Mean IOP and mean number of medications were not statistically different in the phakic and pseudophakic patients at all timepoints (all p>0.05). Overall success and complete success were comparable in the phakic and pseudophakic patients at all timepoints (all p>0.05).

### Adverse events

With regard to safety, complications are shown in **Table 3**.

**Table 3:** Selected safety profiles of the XEN45 and trabeculectomy groups.

Safety Variable	XEN45 (n = 57)	Trabeculectomy (n = 57)	p-value
Early post-operative; n (%) (within 1 month)			
Wound leak	0 (0.0)	9 (15.8)	0.002*
Subconjunctival hemorrhage	5 (8.8)	0 (0.0)	0.022*
Overdrainage	1 (1.8)	4 (7.0)	0.170*
Choroidal detachment	1 (1.8)	1 (1.8)	1.000*
Hyphema	0 (0.0)	1 (1.8)	0.315*
Late post-operative; n (%) (after 1 month)			
Hypotony	1 (1.8)	6 (10.5)	0.041*
Bleb leak	0 (0.0)	3 (6.0)	0.079*
Ptosis	6 (10.5)	2 (3.5)	0.136*
Implant extrusion	3 (5.3)	-	
Device fracture	1 (1.8)	-	
Choroidal detachment	0 (0.0)	1 (1.8)	0.315*
Uveitis	1 (1.8)	1 (1.8)	1.000*

*Chi-square test.

Intraoperatively, subconjunctival hemorrhage occurred in 6 cases (10.5%) and device fracture in 2 cases (3.5%) during XEN implantation. In the trabeculectomy group, 2 cases (3.5%) of flap lacerations, 2 (3.5%) of conjunctival tear, 1 (1.8%) of bleb hemorrhage and 1 case (1.8%) of hyphema were noted.

In the early postoperative phase (within 1 month) [11], wound leak occurred more in the trabeculectomy group, 0 versus 9 eyes (15.8%); p = 0.002. Subconjunctival hemorrhage occurred more in the XEN group (5 eyes (8.8%) versus 0; p = 0.022).

In the later-than-one-month post-operative phase, XEN had a lower rate of numerical hypotony (IOP ≤5 mmHg at ≥1 month apart) than trabeculectomy, at 1 eye (1.8%) versus 6 (10.5%); p = 0.041. Ptosis was noted in 6 eyes (10.5%) in the XEN group and in 2 eyes (3.5%) in the trabeculectomy group; p = 0.136. Conversely, bleb leak was detected in 3 eyes (5.3%) of trabeculectomy patients but none in their XEN counterparts. XEN implant extrusion occurred in 3 eyes (5.3%) and XEN device fracture was detected in 1 eye (1.8%). In the XEN group, 7 patients (12.3%) required SSI throughout the follow-up period, and 4 of these required trabeculectomy with MMC, while 3 had the XEN removed due to implant extrusion. In the trabeculectomy group, 2 patients (3.5%) required SSI which was marginally fewer than the XEN group (p = 0.082), and 1 of these had undergone second trabeculectomy with MMC, while 1 required a tube shunt surgery. During the follow-up period, there was no bleb-related infection, endophthalmitis, suprachoroidal hemorrhage, or serious complication e.g. loss of light perception, after each procedure.

## Discussion

Glaucoma surgery has been evolving in recent years. Newer procedures e.g. Ex-PRESS shunt, viscocanalostomy, deep sclerectomy, trabectome, canaloplasty, i-Stent, Cypass, and Gold shunt have been employed with patients, varying from center to center [12]; however, little is known of the outcomes of these procedures in Asian patients. Asia has the world’s largest population and the number of glaucoma patients appears to be the largest globally [1]. It is estimated that by the year 2040, 66.8 million Asians will have glaucoma. Regarding XEN implant, most studies have been conducted in Western countries with predominately Caucasian participants. There is only one published paper from China [13]. Ethnic differences may present different outcomes; for example, in a comparative study of XEN versus trabeculectomy, being Caucasian was found to be a favorable factor for success [14]. In a non-comparative study in the United Kingdom, Gabbay et al also reported that non-Caucasians had a higher risk of failure of XEN implant [15]. Laroche et al reported that black and Afro-Latin patients needed 40% SSI in 1-year follow up of XEN implantation [16]. XEN gel implant has been available in our practice since 2017, and evaluation of its efficacy and safety is of tremendous importance for our Southeast Asian patients.

In the present study, surgical outcomes showed that overall and complete successes were comparable between XEN and conventional trabeculectomy in the intermediate term of follow up. There have been only a few comparative studies of XEN and trabeculectomy so far. Wagner et al compared XEN and trabeculectomy in Caucasian patients at 12-month follow up, reporting complete success of 58.5 vs 65.5%, respectively, with non-statistical significance using crude and adjusted odd ratios. The complete success rates at 12-month follow up of our patients was 66.7 vs 64.3%, respectively, and it was 62.9 vs 62.2% at 24-month follow up. Schlenker et al [14] conducted a large comparative study of the procedures, enrolling 293 patients (354 eyes: 185 XEN and 169 trabeculectomy) from 4 eye care centers in Canada and Europe, and found that the failure rates of XEN and trabeculectomy were not different. These 3 comparative studies indicate the comparable effectiveness of XEN compared to trabeculectomy in terms of surgical success in different ethnicities.

The present study showed that the XEN mean final IOP at 24-month follow up was higher than that of trabeculectomy, 14.6 vs 12.4 mmHg; this could be observed from month 6 onwards. Many previous studies of XEN achieved a similar result with final IOP of mid-teen level, 14–15 mmHg [9,13–15]. Hu et al reported median final IOP of 15 mmHg (IQR 11–18) in 6-month outcomes of XEN implant in 63 Chinese patients [13]. Two-year results of a large non-comparative study of XEN by Reitsamer et al reported IOP of 15.2 mmHg in 202 implants [9], and Gabbay et al reported 14.5 mmHg in 151 cases [14]. XEN can achieve even lower IOP levels; for example, Wagner et al presented 12-month IOP of 11.8 mmHg in 82 eyes of XEN implantation [17]. In a comprehensive review by Fea et al, final IOP of XEN implant ranged from 12 to 17.1 mmHg in of 8.5 to 24-month follow up [18].

Mid-teen level IOP may be an acceptable target pressure for mild to moderate glaucoma. In Collaborative initial glaucoma treatment study (CIGTS), trabeculectomy achieved average IOP of 14–15 mmHg and the visual field did not change over a 5-year study [5]. In Advanced glaucoma intervention study, maintaining IOP < 18 mmHg at all timepoints in over 6-year follow up can halt the visual filed changes [19]. European Glaucoma Society also recommend IOP < 18 mmHg in moderate stage of glaucoma [20]. In the present research, XEN achieved 32% IOP reduction at the 24-month timepoint. In Early Manifestation Glaucoma Trial (EMGT), average IOP reduction with 360-degree argon laser trabeculoplasty (ALT) and betaxolol was 25%. Glaucoma progression was significantly lower in the treated group than in controls (no treatment) [21], and XEN implant is potentially useful for preventing glaucoma progression in such patients. Both VA and visual field in our XEN group were stable during the follow-up period. XEN implant can be applied for glaucoma in cases of failed medical and laser treatment to achieve mid-teen IOP level and at least 20% IOP reduction.

In general practice for POAG, medications are the first-line treatment. Laser trabeculoplasty and surgery are the next steps considered when medical treatment has failed to control IOP, or in cases of intolerance to medications. Medications need a life-long instillation, and adverse effects and compliance are burdens to patients [22]. Long-term medical instillation has been shown to induce inflammation of conjunctiva and tenon which could affect subsequent surgical success [23,24], Primary trabeculectomy has been debated for decades [25]. Migdal et al reported that primary trabeculectomy in POAG achieved 98% tonometric success (IOP < 22 mmHg) and functional test was stable in 5-year follow up, whereas medical and laser trabeculoplasty achieved 83% and 68% success respectively, with functional test deterioration over time [4]. In Collaborative Initial Glaucoma Treatment Study (CIGTS), a primary trabeculectomy cohort showed lower final IOP than a medical treatment group at 5-year follow up, with stable visual field in both groups [5]. Quality of life after primary surgery might be better than that achieved by chronic medical treatment.

XEN could be an option for primary treatment of glaucoma. First, the evolutions of biocompatible implants and fluid dynamic systems appear to be useful to XEN [26]. Second, the complication rate was acceptable, as no serious complication occurred in the present study. In addition, XEN, similar to other minimally invasive procedures, showed a potential favorable visual outcome and rapid recovery of vision. Our results showed a fairly constant VA throughout the follow-up timepoints in the XEN group with no patient losing 2 Snellen lines equivalent VA. On the other hand, VA was statistically worse than baseline in the trabeculectomy group at month 24 (p = 0.001) with 2 patients losing 2 Snellen lines equivalent VA. The speculative benefits of primary XEN implantation are to be elucidated with a prospective cohort study.

Flat and encapsulated bleb could also be a potential cause of bleb failure in XEN implant. XEN could erode the conjunctiva if the implant touches the conjunctiva of the flat blebs like in 3 cases of this study. Needling revision has been performed to rescue the failing bleb of XEN implant [27]. MMC 20 microgram intra-bleb injection may be added during needling. In our study, the rate of needling was 17.5% in the XEN group and 19.1% in the trabeculectomy group. In other comparative studies of XEN versus trabeculectomy, Schlenker et al performed 43% vs. 31% needling [14] respectively, and Wagner et al performed 16% for both groups [17]. In non-comparative studies, the needling rate ranged from 0–51% [18]. The reason for the variations in needling rates might be related to its diverse indications; in fact, no standard indication has been proposed so far [14,27–31].

Another cause of XEN bleb failure could be related to internal blockade. Descemet detachment during XEN injection, iris, vitreous, or cell debris can block the internal lumen of XEN in the anterior chamber [32]; and gonioscopy should be performed to rule out this condition. Interestingly, we had a case of iris pigment in the lumen of XEN with flat bleb and surgical failure **(S1 Fig)**. Laroche et al. found that iris pigments obstructed XEN lumen and caused surgical failure in their black and Afro-Latin patients [16].

Subgroup analysis comparing POAG and PACG patients who underwent XEN implantation did not show any significant difference at any timepoint up to 24 months with the same baseline characteristics. The mean percent IOP reduction seemed to be lower in PACG than in POAG group at 24 months, but no statistical significance was detected; this might be due to the relatively small number of subjects in the PACG group. In addition, standalone XEN showed comparable outcomes to those of Phaco+XEN, and this was also possibly related to the small number of subjects.

### Complications and management

In terms of safety, XEN showed a favorable safety profile compared with trabeculectomy with no serious complication occurring up to the 24-month follow-up. XEN also had a lower rate of numerical hypotony (IOP ≤5 mmHg at ≥1 month apart) and bleb leak than trabeculectomy. Most complications were transient and self-limited in both groups.

There were 2 remarkable complications in the XEN implant group which have rarely been mentioned in previous studies: ptosis and nasal bleb. Six eyes (10.5%) had marked ptosis and did not improve over time **(S2 Fig)**. Ptosis might be related to the following causes. First, the Asian interpalpebral fissure is relatively small, and trying to open the lid widely for surgical field with lid speculum could injure the levator aponeurosis. Second, MMC and xylocaine injection at the superior nasal conjunctiva might spread to the superior rectus muscle and be toxic to it. Even though low doses of MMC were used, it could not be washed out as we could in trabeculectomy. The remaining MMC and xylocaine would last longer inside the injected area and might have toxic effects on the superior rectus and levator muscles. As yet, there is no standard dose of MMC for XEN implantation. In management, we observed those cases for 6 months. Two cases underwent subsequent ptosis correction by an oculoplastic surgeon.

Nasal bleb was another complication and occurred in 5 eyes (8.8%) of the XEN group. Patients presented with large cystic bleb extended from the superior to the nasal conjunctival area. Epiphora and Dellen cornea were observed. Nasal bleb could have occurred along with the spread of MMC subconjunctival injection. This symptomatic bleb was tapped with a 30-G needle, but it recurred later. Repeated tapping was performed upon the bleb size and symptoms. Yavuzer demonstrated a technique using a drainage channel with sutures to manage this complication [33]; however, surgical techniques for avoiding this problem are not yet available.

### Study limitations

The non-randomized, retrospective nature of this study may have inherently created information and selection bias. Although the clinical data from the chart represent real-world results, uncontrollable factors, such as patients being lost to follow-up and missing information, may have created statistical errors. This study also included both phakic, pseudophakic and combined Phaco+XEN patients in which IOP reduction might be related to cataract extraction. The subgroup of PACG was of relatively small size, so that some differences may not show statistically significant results. The ethnicity in this study was also limited to a Southeast Asian population. Surgeon factor might be another limitation since both procedures were not performed by the same surgeon. However, we believe that this manner is a standard practice in many tertiary eye care training centers. Also, since both eyes were enrolled in 5 patients, possible crossover effect from the use of beta-blocker eye drops might also affect the IOP lowering results. Issues of cornea endothelial cell counts, bleb morphology, quality of life and cost effectiveness were not determined within the study, and assessment of a long-term follow up period of 3 to 5 years is essential for this new implant. Further prospective, multicenter, multi-ethnicity, randomized, controlled trials are required to verify the efficacy and safety of the procedure.

## Conclusions

Our study presents intermediate term real-world surgical outcomes of XEN implantation in Asian eyes compared with trabeculectomy. At 24-month follow up, XEN showed a rate of success comparable to that of trabeculectomy. Although XEN had a higher final IOP than trabeculectomy, XEN achieved 32% IOP reduction, and achieved final IOP in mid-teen level. No serious complication occurred in either group. XEN can be applied for treatment of mild to moderate stages of primary glaucoma in Asian patients.

## Supporting information

S1 Fig Iris pigment in the internal lumen of kinked XEN implant with flat bleb and surgical failure. This patient underwent a second surgical intervention (trabeculectomy) afterward.
(TIF)

S2 Fig Marked ptosis of the left eye persisted after XEN implantation. Patient underwent oculoplastic ptosis correction 6 months postoperatively.
(TIF)

S1 Table Characteristics of XEN45 and trabeculectomy in phakic and pseudophakic patients. (DOCX)

S1 File (SAV)
