# Supplementary figures and images for: 24-month outcomes of XEN45 gel implant versus trabeculectomy in primary glaucoma
Source: PLoS One. 2021 Aug 19;16(8):e0256362. doi: 10.1371/journal.pone.0256362 (PMC8376039; doi:10.1371/journal.pone.0256362)

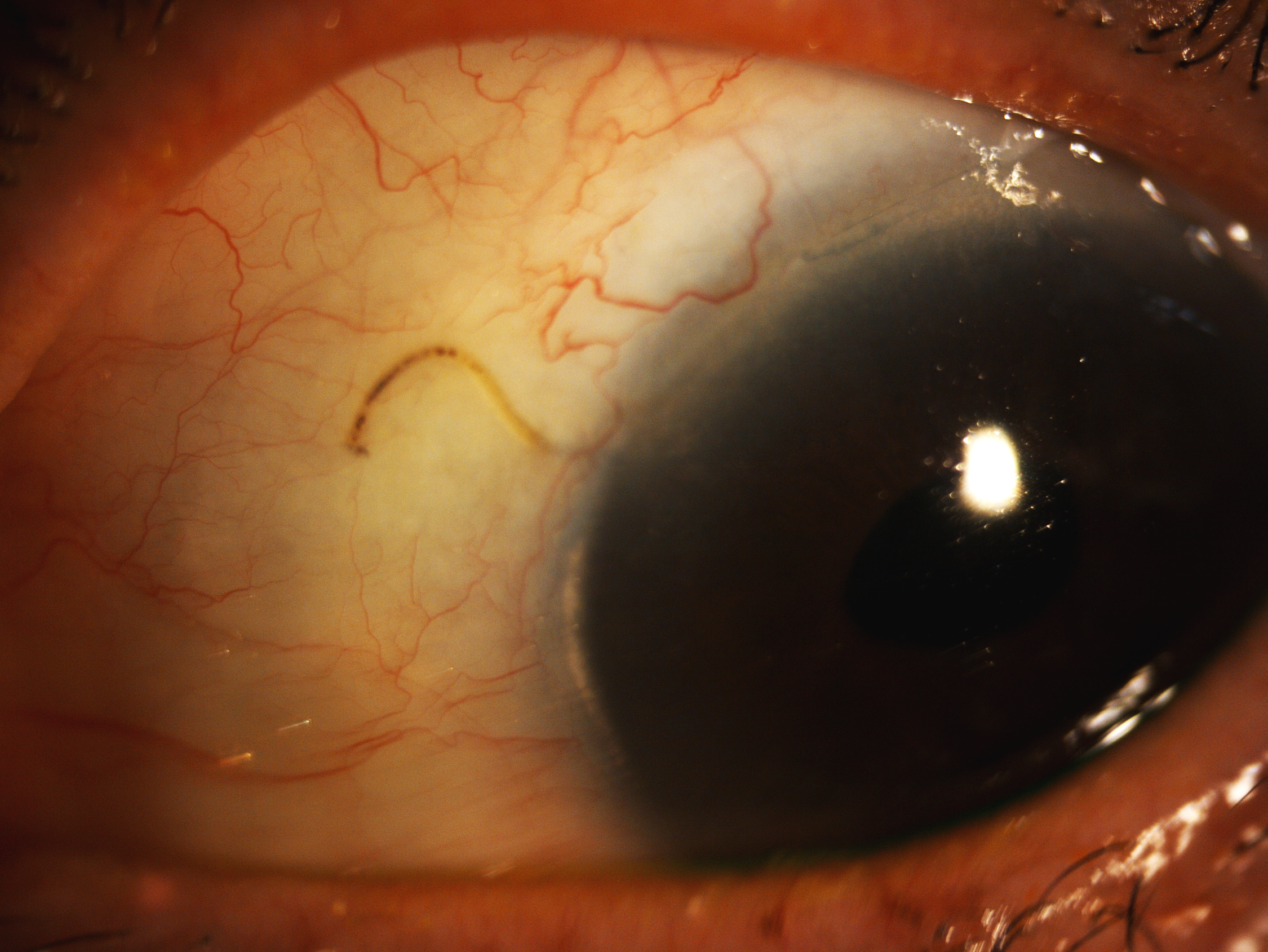

Supplement: S1 Fig — This patient underwent a second surgical intervention (trabeculectomy) afterward. (TIF) [file pone.0256362.s001.tif]

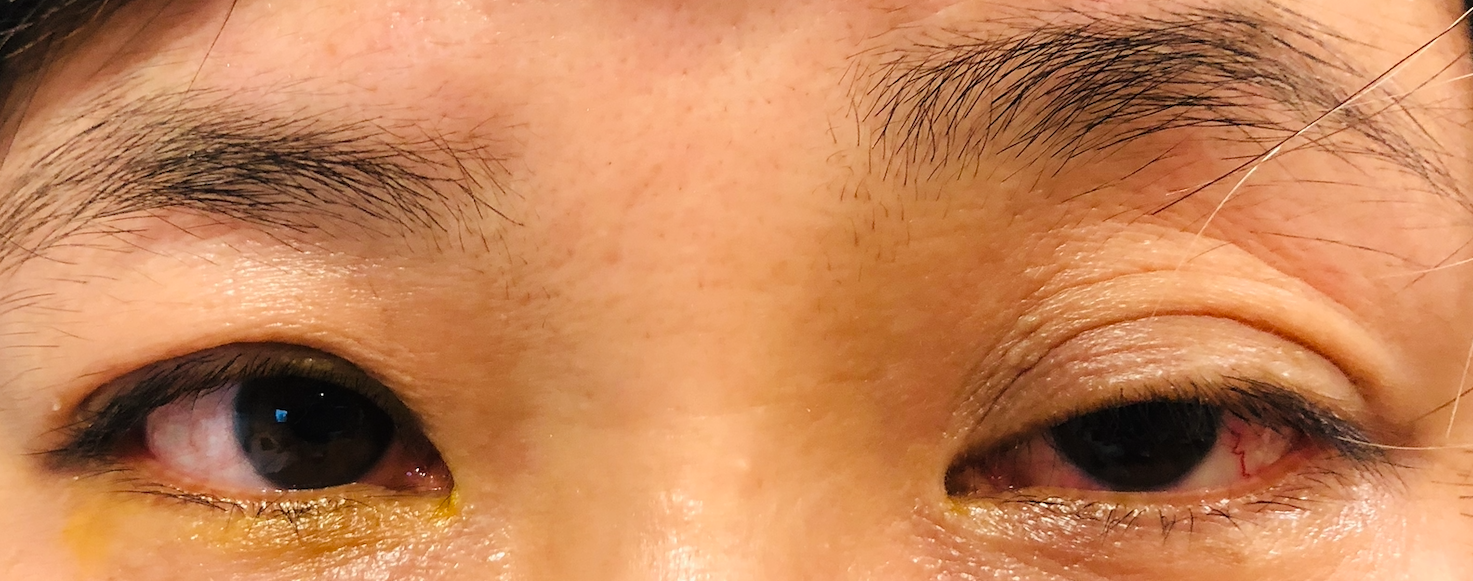

Supplement: S2 Fig — Patient underwent oculoplastic ptosis correction 6 months postoperatively. (TIF) [file pone.0256362.s002.tif]
