# Supplementary material for: 24-month outcomes of XEN45 gel implant versus trabeculectomy in primary glaucoma
Source: PLoS One. 2021 Aug 19;16(8):e0256362. doi: 10.1371/journal.pone.0256362 (PMC8376039; doi:10.1371/journal.pone.0256362)
Supplement: S1 Table — (DOCX) [file pone.0256362.s003.docx]

**S1 Table** Characteristics of XEN45 and trabeculectomy in Phakic and Pseudophakic patients

| **Variable** | **XEN45** | | **p-value** | **Trabeculectomy** | | **p-value** |
| --- | --- | --- | --- | --- | --- | --- |
|  | **Phakic (n=16)** | **Pseudophakic (n=37)** |  | **Phakic (n=30)** | **Pseudophakic (n=27)** |  |
| Gender; n (%) |  |  | 0.945* |  |  | 0.483* |
| Male | 8 (50.0) | 17 (45.9) |  | 15 (50.0) | 16 (59.3) |  |
| Female | 8 (50.0) | 20 (54.1) |  | 15 (50.0) | 11 (40.7) |  |
| Age (year) | 64.1 ± 7.2 | 73.9 ± 7.0 | <0.001^†^ | 64.8 ± 7.0 | 73.5 ± 7.8 | <0.001^†^ |
| VA (decimal) | 0.69 ± 0.28 | 0.66 ± 0.21 | 0.655^†^ | 0.54 ± 0.27 | 0.46 ± 0.26 | 0.295^†^ |
| Refraction (SE) | -1.06 ± 2.75 | -0.22 ± 0.60 | 0.196^‡^ | -1.14 ± 2.71 | -0.63 ± 2.16 | 0.436^‡^ |
| CCT (µm) | 523.3 ± 8.8 | 524.4 ± 4.3 | 0.506^†^ | 532.3 ± 9.8 | 534.1 ± 3.7 | 0.371^†^ |
| Axial length (mm) | 23.79 ± 0.86 | 23.33 ± 0.53 | 0.062^†^ | 23.84 ± 1.01 | 23.68 ± 1.07 | 0.568^†^ |
| Diagnosis; n (%) |  |  | 0.642* |  |  | 0.205* |
| POAG | 13 (81.3) | 28 (75.7) |  | 20 (66.7) | 22 (81.5) |  |
| PACG | 3 (18.7) | 9 (24.3) |  | 10 (33.3) | 5 (18.5) |  |
| Cup-to-disc ratio | 0.71 ± 0.12 | 0.72 ± 0.12 | 0.664^†^ | 0.77 ± 0.13 | 0.78 ± 0.15 | 0.824^†^ |
| Mean deviation (dB) | -10.96 ± 7.86 | -8.11 ± 6.26 | 0.140^‡^ | -8.78 ± 5.70 | -10.67 ± 4.12 | 0.163^‡^ |
| Mean number of medications | 2.2 ± 1.3 | 2.2 ± 1.4 | 0.852^†^ | 2.4 ± 0.7 | 2.4 ± 0.8 | 0.763^†^ |
| Mean IOP (mmHg) | 21.3 ± 4.1 | 21.9 ± 3.9 | 0.152^†^ | 22.3 ± 5.8 | 22.7 ± 6.0 | 0.798^†^ |
| Mean IOP at month 3 (mmHg) | 14.6 ± 6.6 | 13.2 ± 5.0 | 0.128^†^ | 12.3 ± 5.0 | 13.1 ± 4.4 | 0.543^†^ |
| Mean IOP at month 6 (mmHg) | 14.8 ± 4.3 | 13.8 ± 4.0 | 0.380^†^ | 11.2 ± 3.5 | 12.8 ± 5.0 | 0.150^†^ |
| Mean IOP at month 12 (mmHg) | 15.6 ± 4.0 | 14.7 ± 4.7 | 0.513^†^ | 12.8 ± 4.1 | 12.9 ± 4.2 | 0.929^†^ |
| Mean IOP at month 18 (mmHg) | 15.4 ± 4.7 | 13.9 ± 3.2 | 0.165^†^ | 12.3 ± 4.2 | 13.6 ± 6.2 | 0.366^†^ |
| Mean IOP at month 24 (mmHg) | 15.0 ± 2.9 | 14.4 ± 3.4 | 0.499^†^ | 12.3 ± 3.6 | 12.6 ± 4.3 | 0.809^†^ |
| Mean number of medications at month 3 | 0.7 ± 1.3 | 0.4 ± 1.0 | 0.203^†^ | 0.3 ± 1.1 | 0.4 ± 1.0 | 0.789^†^ |
| Mean number of medications at month 6 | 0.7 ± 1.2 | 0.4 ± 0.9 | 0.303^†^ | 0.4 ± 1.2 | 0.7 ± 1.4 | 0.309^†^ |
| Mean number of medications at month 12 | 0.6 ± 0.8 | 0.5 ± 1.0 | 0.627^†^ | 0.6 ± 1.2 | 0.9 ± 1.4 | 0.389^†^ |
| Mean number of medications at month 18 | 0.6 ± 0.8 | 0.5 ± 0.9 | 0.749^†^ | 0.8 ± 1.4 | 1.0 ± 1.4 | 0.713^†^ |
| Mean number of medications at month 24 | 0.6 ± 0.7 | 0.5 ± 0.8 | 0.433^†^ | 0.9 ± 1.4 | 0.9 ± 1.3 | 0.972^†^ |
| Overall success at month 3 (%) | 75.0 | 83.3 | 0.452* | 83.3 | 74.1 | 0.392* |
| Overall success at month 6 (%) | 75.0 | 81.8 | 0.553* | 80.0 | 77.8 | 0.837* |
| Overall success at month 12 (%) | 75.0 | 78.6 | 0.772* | 75.0 | 74.1 | 0.937* |
| Overall success at month 18 (%) | 70.0 | 75.0 | 0.711* | 74.1 | 73.1 | 0.934* |
| Overall success at month 24 (%) | 70.0 | 72.0 | 0.883* | 75.0 | 71.4 | 0.787* |
| Complete success at month 3 (%) | 65.0 | 72.2 | 0.573* | 73.3 | 66.7 | 0.583* |
| Complete success at month 6 (%) | 65.0 | 75.8 | 0.399* | 73.3 | 66.7 | 0.583* |
| Complete success at month 12 (%) | 60.0 | 71.4 | 0.408* | 67.9 | 62.9 | 0.840* |
| Complete success at month 18 (%) | 60.0 | 70.8 | 0.450* | 66.7 | 61.5 | 0.697* |
| Complete success at month 24 (%) | 60.0 | 65.0 | 0.783* | 66.7 | 57.1 | 0.511* |

*Chi-square test, †Student’s t-test, ‡Mann-Whitney U test
